# Endovascular management of renal artery aneurysms induced by neurofibromatosis type 1: A case report

**DOI:** 10.1097/MD.0000000000008858

**Published:** 2017-11-27

**Authors:** Bihui Zhang, Yinghua Zou, Min Yang, Guochen Niu

**Affiliations:** Department of Interventional Radiology and Vascular Surgery, Peking University First Hospital, Peking University, Beijing, China.

**Keywords:** embolization, endovascular procedure, hypertension, neurofibromatosis type 1, renal artery aneurysms

## Abstract

**Rationale:**

Neurofibromatosis type 1 (NF-1) is an autosomal dominant disorder characterized by cafe au lait macules and neurofibromatosis. Renal artery aneurysms are relatively uncommon. Endovascular techniques are effective in treating renal aneurysms but successful cases are rarely reported in NF-1 adults.

**Patient concerns:**

The patient was one 23-year-old female presented with hypertension, multiple café-au-lait spots ≥15 mm, and plexiform neurofibroma. Renal artery aneurysms were found by ultrasound.

**Diagnoses:**

NF-1 was diagnosed based on clinical manifestations and confirmed by gene test. Renal artery aneurysms were diagnosed based on computed tomography.

**Interventions:**

Bilateral renal artery angiography was performed and 3 aneurysms were found sequentially on the left anterior superior segmental artery. Microcoil embolization of aneurysms was undertaken.

**Outcomes:**

The patient's blood pressure decreased after the procedure with reduction of medicine. A 3-month follow-up unilateral selective renal angiogram demonstrated little change in size of aneurysms, and no opacification of the aneurysmal sac was found. Serum creatinine remained in normal range at 3-month.

**Lessons:**

Successful endovascular treatment for NF-1 related renal artery aneurysms in adults is reported for the 1st time with preserved renal function and improved hypertension. Endovascular procedure is considered to be feasible and effective for renal artery aneurysms induced by NF-1.

## 1 Introduction

Neurofibromatosis type 1 (NF-1), previously known as von Recklinghausen neurofibromatosis or peripheral neurofibromatosis, is an autosomal dominant disorder affecting about 1 in 4000 individuals.^[1,2]^ Characteristic manifestations are cafe au lait macules and neurofibromas.^[1]^ Patients with NF-1 may also develop cardiovascular diseases.^[3]^ NF-1 vasculopathy includes vascular stenosis, occlusion, aneurysm, pseudoaneurysm, rupture, or fistula formation and is usually recognized in childhood or early adulthood, often during pregnancy.^[3,4]^ Hypertension is common among NF-1 patients, may be essential or secondary to renovascular disease, coarctation of the aorta, or phaeochromocytoma.^[5]^ Renal arteries are most frequent sites of symptomatic vasculopathy but most were stenotic lesions rather than aneurysms.^[3,6]^ Aneurysms associated with NF-1 treated by endovascular procedures were rarely reported.^[6]^ In the present report, we describe a case of an adult female NF-1 patient presented with hypertension and renal artery aneurysms, who was treated successfully by endovascular procedure.

## 2 Case report

A 23-year-old woman came to the hospital with blood pressure of about 150/100 mm Hg while taking Valsartan amlodipine (80 mg/5 mg) twice daily. She denied involuntary movements, blurry vision, headaches, or neurological deficits. She had a history of severe preeclampsia (blood pressure higher than 180/120 mm Hg) at 20 weeks of gestation and took artificial abortion. Her family history of hypertension or cardiovascular disease was unremarkable.

**Figure 1:**
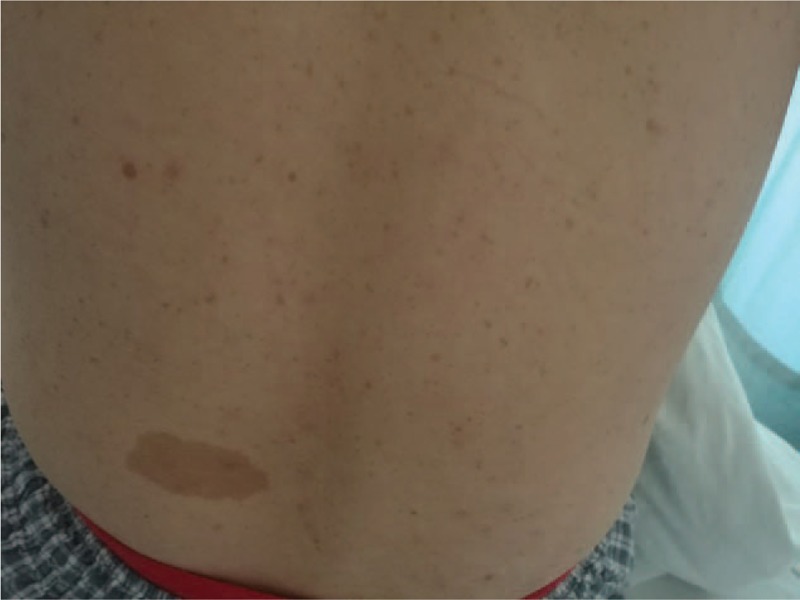
Multiple café-au-lait spots ≥15 mm on the patient's back.

Upon physical examination, orbital hypertelorism was found and multiple café-au-lait spots ≥15 mm were revealed all over the whole body (Fig. 1). Plexiform neurofibroma was found over the skin of left upper arm. NF-1 was diagnosed because more than 2 of 7 clinical diagnostic criteria for NF-1 were met.^[1]^

**Figure 2:**
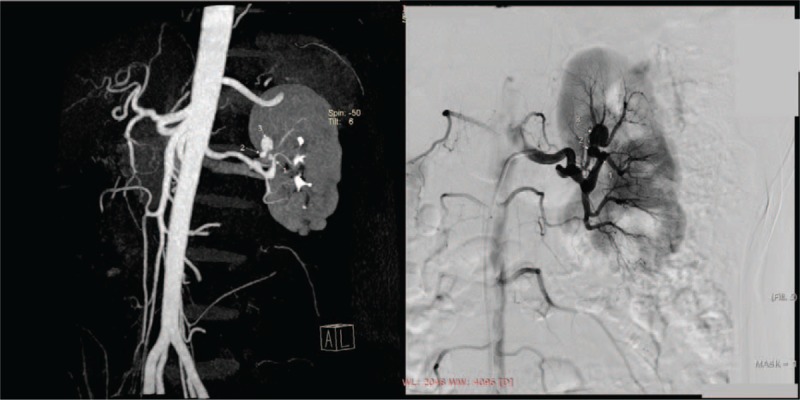
Computed tomography (2A) and angiography (2B) displayed three intrarenal aneurysms of the left kidney.

Previous ultrasound reported no abnormality in bilateral renal arteries and smaller left kidney (right 11.0 × 5.4 × 3.8 cm; left 9.1 × 4.5 × 4.0 cm). Computed tomography angiography revealed left renal artery aneurysms and left kidney shrinkage (Fig. 2A). Brain magnetic resonance image and electroencephalogram were normal and no Lisch nodules were found. Renal Tc 99-m diethylene triamine pentaacetic acid scintigraphy showed impaired left kidney glomerular filtration (glomerular filtration rate: left 18.64 mL/min; right 47.44 mL/min). Renin, aldosterone levels, and renin/aldosterone ratios were all in normal range in both supine and erect positions.

Bilateral renal artery angiography was performed in our catheterization laboratory and 3 aneurysms were found sequentially on the left anterior superior segmental artery (Fig. 2B). Microcoil embolization of aneurysms was undertaken and the left kidney perfusion was not compromised on postprocedure angiography.

**Figure 3:**
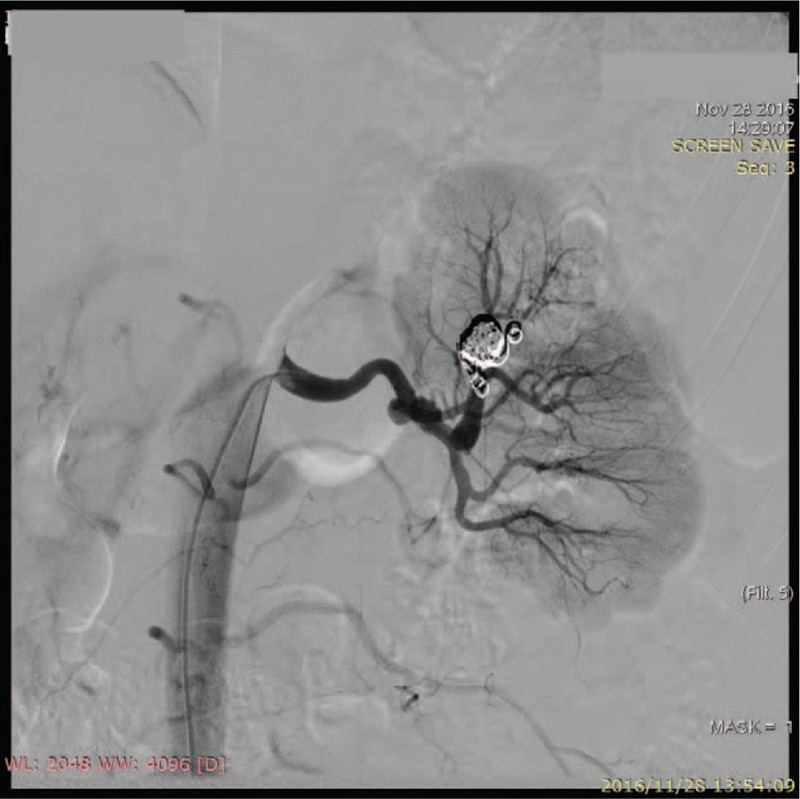
Angiography 3 months after the embolization of intrarenal aneurysms.

**Figure 4:**
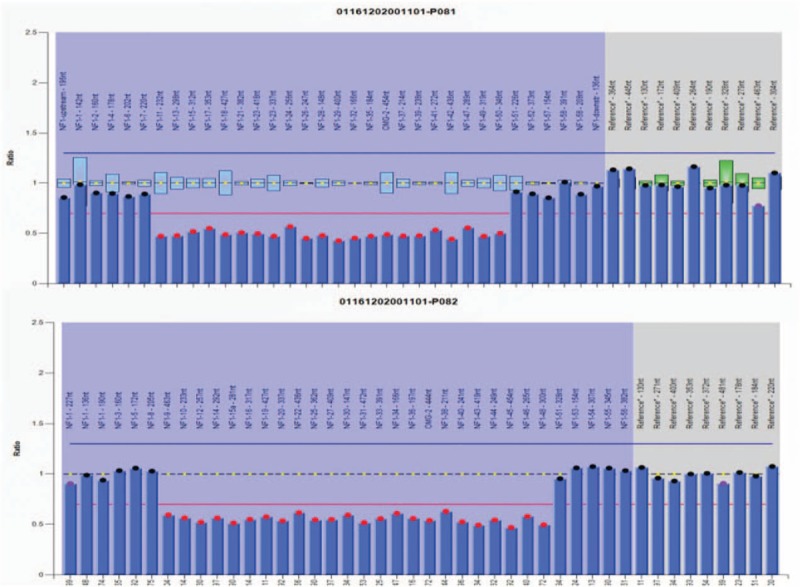
Identification of large NF-1 deletions containing exons 9–50 via multiplex ligation-dependent probe amplification (MLPA).

The patient's blood pressure reduced to 110/60 mm Hg next morning after the procedure without antihypertensive medicine and maintained for a month with Valsartan amlodipine (80 mg/5 mg) once daily. Then her blood pressure increased to 130–140/80–90 mm Hg with Valsartan amlodipine (80 mg/5 mg) once daily for until 3-month follow-up. A 3-month follow-up unilateral selective renal angiogram demonstrated little change in size of aneurysms and no opacification of the aneurysmal sac was found (Fig. 3). Serum creatinine remained in normal range at 3-month. Large NF-1 deletions containing exons 9–50 were identified via multiplex ligation-dependent probe amplification (Fig. 4). The patient was advised to access genetic counseling during early pregnancy.

## 3 Discussion

NF-1 is an autosomal dominant disorder and NF-1 gene is localized at chromosome 17q11.2.^[1,7]^ Almost 50% of all affected individuals have de novo mutation. NF-1 has a complete penetrance, but each affected individual presents with some of the widely variable disease manifestations.^[8]^ People with NF-1 can develop cardiac and vascular disease, which is known as NF-1 vasculopathy.^[3]^ But the frequency, natural history, and pathogenesis are uncertain because screening is not routine and many patients are asymptomatic throughout life.^[9]^ The patient in the present case is the proband of the family and is considered to have de novo mutation because neither of her parents has manifestations of NF-1.

Hypertension, including essential and secondary, is present in about 6% of patients with NF-1.^[5]^ Renal artery stenosis is a common cause of secondary hypertension and is usually observed in childhood or young adulthood, especially during pregnancy.^[3,10]^ Open or endovascular techniques, nephrectomy or medication have been used to treat renovascular hypertension in NF-1 patients, but ineffectiveness and recurrence often occur.^[6]^ Fewer aneurysmal than stenotic renal artery lesions are seen among NF-1 vasculopathies.^[6]^ Renal artery aneurysms may be unilateral or bilateral with or without calcification, and rupture may occur spontaneously without risk factors in NF-1 patients.^[11–13]^ Endovascular treatment for NF-1 related renal artery aneurysms is rarely reported. Triantafyllidi et al^[14]^ described the 1st percutaneous transluminal renal angioplasty to restore renal artery anatomy and treat hypertension in an adult female patient. But hypertension recurred and the treated kidney revealed no function 1 month after the procedure.^[14]^ One child associated with 3 small aneurysms was treated by superselective embolization, and her blood pressure was normalized without medication.^[15]^ In this case, successful endovascular treatment for NF-1 related renal artery aneurysms in an adult is reported for the 1st time with preserved renal function and improved hypertension. Endovascular procedure is considered to be feasible and effective for renal artery aneurysms induced by NF-1 by the authors.

NF-1 is characterized by highly variable clinical expressivity with marked inter- and intrafamilial variation in both the number of major features and the occurrence of complications.^[16]^ The allelic heterogeneity of the constitutional NF-1 mutation may explain the phenotypic variability. Large deletions of the NF-1 gene region were found in about 5% of all NF-1 patients and have been described to be disproportionately associated with facial dysmorphic features, plexiform neurofibromas, subcutaneous neurofibromas, spinal neurofibromas, and mental retardation.^[17]^ For intragenic NF-1 mutations, no clear-cut allele–phenotype correlations have been established so far, with the exception of a 3-bp in-frame deletion (c.2970–2972 delAAT) in exon 17 of the NF-1 gene, which was associated with a particular clinical phenotype characterized by the absence of cutaneous neurofibromas.^[7,18]^ Genetic counseling should be offered to young adults who are affected or at risk and prenatal genetic testing is essential during pregnancy.

## 4 Conclusion

NF-1 is autosomal dominant disorder and can cause hypertension and renal artery aneurysms that are difficult for surgeries. Endovascular procedure is a feasible therapy and prognosis is favorable.
